# Identification and Characterization of a Spore-Like Morphotype in Chronically Starved *Mycobacterium avium* Subsp. *Paratuberculosis* Cultures

**DOI:** 10.1371/journal.pone.0030648

**Published:** 2012-01-24

**Authors:** Elise A. Lamont, John P. Bannantine, Aníbal Armién, Don Sanjiv Ariyakumar, Srinand Sreevatsan

**Affiliations:** 1 Veterinary Population Medicine, University of Minnesota, St. Paul, Minnesota, United States of America; 2 Department of Veterinary Biomedical Sciences, University of Minnesota, St. Paul, Minnesota, United States of America; 3 Veterinary Diagnostic Lab, University of Minnesota, St. Paul, Minnesota, United States of America; 4 Agricultural Research Service, National Animal Disease Center, United States Department of Agriculture (USDA), Ames, Iowa, United States of America; Universita di Sassari, Italy

## Abstract

Mycobacteria are able to enter into a state of non-replication or dormancy, which may result in their chronic persistence in soil, aquatic environments, and permissive hosts. Stresses such as nutrient deprivation and hypoxia provide environmental cues to enter a persistent state; however, a clear definition of the mechanism that mycobacteria employ to achieve this remains elusive. While the concept of sporulation in mycobacteria is not novel, it continues to spark controversy and challenges our perceptions of a non-replication. We investigated the potential role of sporulation in one-year old broth cultures of *Mycobacterium* subsp. *paratuberculosis* (*MAP*).

We show that dormant cultures of *MAP* contain a mix of vegetative cells and a previously unknown morphotype resembling a spore. These spore-like structures can be enriched for using sporulating media. Furthermore, purified *MAP* spore forms survive exposure to heat, lysozyme and proteinase K. Heat-treated spores are positive for *MAP 16SrRNA* and *IS900*. *MAP* spores display enhanced infectivity as well as maintain acid-fast characteristics upon germination in a well-established bovine macrophage model. This is the first study to demonstrate a new *MAP* morphotype possessing spore-like qualities. Data suggest that sporulation may be a viable mechanism by which *MAP* accomplishes persistence in the host and/or environment. Thus, our current understanding of mycobacterial persistence, pathogenesis, epidemiology and rational drug and vaccine design may need to be reevaluated.

## Introduction

Mycobacteria represent a group of highly successful organisms that range from free-living saprophytes to those that have adapted full dependence on a living host [1], [2]. During their life cycle, mycobacterial species may encounter a number of stresses including nutrient deprivation, hypoxia, acidic pH, and even competition with other organisms for limited resources and occupation of a specific niche, such as soil and water [3], [4], [5], [6]. In order to survive in such unfavorable conditions, mycobacteria have developed mechanisms to achieve dormancy, latency and persistence [7], [8], [9]. While several studies have investigated persistence in mycobacteria, the definition remains loosely explained and the mechanisms that lead to and sustain this state of non-replication are poorly understood. A recent study by Ghosh et al. stated the formation of endospores in two month old cultures of *M. marinum* and *M. bovis*, which may serve as an unprecedented method employed by mycobacteria to withstand harsh conditions [10]. The concept of sporulation in mycobacteria continues to spark controversy and challenges our current perceptions of the facets involved in mycobacterial persistence. Follow-up studies conducted by Traag et al. could not reproduce endospore formation in 4 week to ∼8.5 month liquid cultures of *M. marinum*, which questioned the purity of cultures used in ultrastructural characterization by Ghosh et al. [11]. The current research trend focusing on sporulation in mycobacteria remains to reproduce findings by Ghosh et al. using identical isolation methods; however, we investigated whether the potential for sporulation was limited to *M. marinum* or may encompass another saprophyte and animal pathogen, *Mycobacterium avium* subsp. *paratuberculosis* (*MAP*).

*MAP*, the causative agent of Johne's disease (JD) in ruminants, is one of the most prevalent and well-documented infections of dairy cattle worldwide [12]. To date, JD eradication remains implausible and control efforts are hampered due to *MAP*'s persistence within soil and water as well as shedding by subclinical and clinical cattle [12], [13], [14], [15]. Therefore, it is critical to augment our knowledge of the events that precede non-replication as well as the various mechanisms used to attain it. Our data showed that one year old broth cultures of *MAP* strains *K-10*, *7565*, *Linda* and *Ben* have the potential to produce a previously undocumented morphotype possessing a spore-like structure given optimal sporulation conditions. All isolated *MAP* spore-like morphotypes shared appropriate spore ultrastructures, presence of dipicolinic acid and the *MAP* specific insertion sequence, *IS900*, and heat resistance. More importantly, heat treated *MAP* spore structures retained macrophage infectivity as well as acid-fast characteristics upon germination. These data suggest that sporulation may be a viable route by which *MAP* accomplishes persistence in the environment.

## Results and Discussion

### MAP produces a new morphology when grown on Arret-Kirshbaum sporulation agar under physiologically relevant temperature

**Figure 1:**
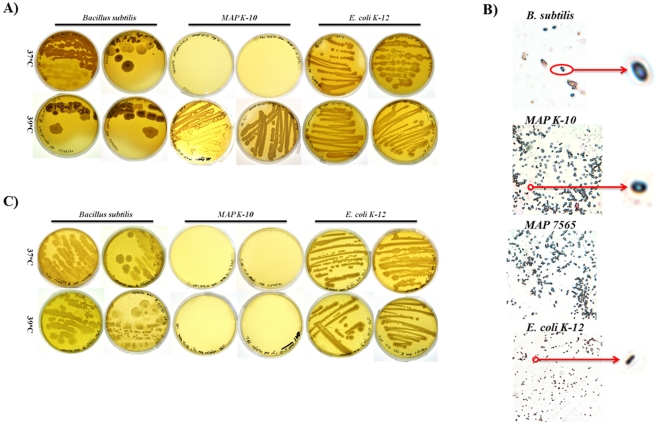
*MAP* morphotype induction is dependent upon temperature. One year old MB7H9 *MAP* broth cultures were inoculated on A) A–K agar and C) BHI agar for 72 h at 37°C and 39°C. *MAP* showed growth only at 39°C compared with *B. subtilis* and *E. coli K-12* controls, which had substantial growth at both temperatures (A). Spore enrichment was determined by malachite green staining (B). In order to confirm purity of *MAP* culture, the year old *MAP* culture was grown on BHI agar and determined to be free of any contaminating organisms (C).

One year old Middlebrook 7H9 (MB7H9) broth cultures (herein referred to as dormant cultures) of *MAP* strains *K-10* (cattle isolate) and *7565* (sheep isolate) were used to examine sporulation activity and isolate spores. It is important to note that the culture medium was never changed and agitation was not supplied for ∼6 months (mo.); therefore, *MAP* strains were assumed to be subject to nutrient starvation and hypoxia. These methods differ from those utilized by Ghosh et al. in which *M. marinum* and *M. bovis* were grown on 7H10 agar plates for 2 and 12 weeks, respectively [10]. Methods also varied from those described by Traag et al. since *MAP* broth cultures were kept at a constant 37°C as opposed to intermittent incubations at 4°C and 33°C. Furthermore, we included a spore enrichment process in which *MAP* broth cultures were separately plated on Arret-Kirshbaum (A–K) sporulating agar and grown at 37°C and 39°C. Since A–K sporulating agar is used for induction of sporulation, particularly from *Bacillus spp.*, we hypothesized that *MAP* may also produce spores upon nutrient exhaustion in this medium. We showed that *MAP* is capable of growth and sporulation on A–K agar after 72 h of incubation at 39°C, which was validated by differential staining for spores using malachite green and safranin (Figure 1A and B). These results are in stark contrast to *E. coli K-12* A–K growth, which is negative for malachite green staining (Figure 1B). It is interesting to note that successful *MAP* growth on A–K medium occurred at 39°C and not at 37°C, the physiological body temperature of cattle in contrast to the *B. subtilis* control which grew at 37°C and 39°C (Figure 1A). Previous studies from our laboratory demonstrate that physiologically relevant temperatures greatly influence *MAP* gene expression profiles and speed of macrophage invasion [16]. It is well recognized that mycobacteria are sensitive to changes in temperature, which influence growth, cell morphology and pathogenesis [16], [17], [18], [19]. Temperature also impacts sporulation efficiency [20], [21], [22]. As previously mentioned, dormant cultures of *M. marinum* used by Traag et al. were stored at 4°C for an unspecified period of time, which may provide one explanation to the lack of spore detection [11]. Thus, additional pressures like host related temperature may be one of several contributing stressors capable of inducing differential rates of sporulation.

Due to the age of *MAP* cultures, a valid concern arose that isolated morphotypes were not of a *MAP* origin but a known endospore contaminant. In order to confirm *MAP* purity, dormant *MAP K-10* cultures used for spore enrichment were streaked on Brain-Heart Infusion (BHI) agar. While *B. subtilis* produced visible colonies, *MAP K-10* showed no growth and was determined to be devoid of any contaminating organisms (Figure 1C). Also, *MAP K-10* was revived on MB7H9 agar indicating that dormant cultures remained viable. Therefore, *MAP K-10* has the potential to produce spore-like structures given sufficient and appropriate cues. Environmental cues that are involve nutrient and moisture limitation, temperature, hypoxia, and competing microbes are hypothesized to be sufficient to cause mycobacterial sporulation but may not all be necessary at the same time.

### Transmission Electron Microscopy (TEM) shows presence of spore-like forms

**Figure 2:**
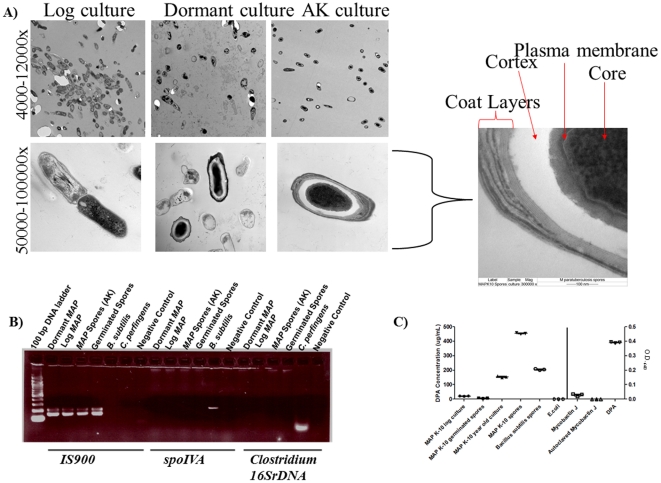
Ultrastructural characterization of *MAP* morphologies. Fine *MAP* morphotype structure was determined by TEM (A). TEM images were taken of log-phase, dormant and A–K *MAP* cultures. While the dormant *MAP* culture showed a mix of vegetative cells and spores, A–K *MAP* cultures displayed typical spore characteristics, including a cortex, plasma membrane and coat layers. (B) All *MAP cultures* were assessed for contamination of duplex and normal PCR of *IS900*, *spoIVA* and *Clostridium 16SrDNA.* Only *MAP* samples contained the *IS900* element and did not amplify *Bacillus* and *Clostridium* related genes. (C) Spore formation was confirmed by the detection of dipicolnic acid (DPA) using a colorimetric assay. DPA is a chemical found within the spore core of endospores. Intact and autoclaved mycobactin J (250.0 µg/mL) were used as controls. Each sample was conducted in triplicate.

Since malachite green primarily functions by permeating the mother cell through heat and is retained within the cell coat layers due to their thickness, the possibility remained that malachite green may be bound to the complex, waxy mycobacterial cell wall. In fact, malachite green has been used to stain flagella and leprosy bacilli in addition to endospores [23], [24]. To validate A–K agar and malachite green staining results and characterize the *MAP* spore-like structure, we examined the spore forms during different phases of growth: log, dormant, and induced (A–K isolated) spore phases (Figure 2A). Dormant *MAP K-10* cultures displayed a mix of bacilli and spores, while the induced phase contained only spores (Figure 2A). Next, we increased the stringency of our testing for possible contamination and conducted duplex and normal polymerase chain reactions (PCRs) to demonstrate the presence and/or absence of *IS900* integration sites in *MAP*, *Bacillus spoIVA* gene and *Clostridium 16SrDNA* gene in all *MAP* and control samples (Figure 2B). *MAP* dormant, log phase, germinated and A–K isolated spore cultures showed the presence of *IS900* integration sites but did not display amplification of specific *Bacillus* and *Clostridium* gene elements, which suggested the unlikelihood of isolated *MAP* morphotypes containing *Bacillus* and *Clostridium* species (Figure 2B). As expected, control spore samples from *B. subtilis* and *C. perfringens* did not amplify the *MAP* specific *IS900* integration sites (Figure 2B).

We tested if *MAP* dormant and A–K spore cultures represented true spores and not a simple thickening of the cell wall as has been reported by Cunningham et al. by determining the presence of dipicolinic acid (DPA; pyridine-2,6 dicarboxylic acid), a chemical commonly found in spores [25]. Calcium bound DPA is found within the spore core and contributes to spore DNA resistance to wet and dry heat, desiccation and hydrogen peroxide [26], [27], [28]. In order to detect DPA, we used a colorimetric assay that releases DPA after autoclaving and acetic acid treatment and is subsequently available to bind to iron under an acidic pH [29]. Since mycobacteria secrete, contain, and are supplemented with siderophores, which form complexes with iron, we included a mycobactin J control to ensure abrogation of potential reactions with iron involved in DPA detection (Figure 2C) [30]. Elevated levels of DPA were found in *MAP* dormant and spore cultures and *B. subtilis* spores (matched for wet pellet weight) (Figure 2C). Not surprisingly, isolated *MAP* spores contained increased amounts of DPA (470.0 µg/mL) compared to any other *MAP* culture, which was not due to reactive mycobactin J (Figure 2C). It has been noted that DPA synthesis in addition to endospore formation occurs with low guanine (G) and cytosine (C) % content among Firmicutes, which conflicts with the high percentage of G+C found in mycobacteria [11]. Although rare, members of the *Streptomyces* (*S. globisporus* and *S. avermitilis*) are capable of endospore production given conditions conducive to endsporulation such as incubation in submerged cultures and phosphate limitation [31], [32]. Also, the presence of DPA was found by Stastná et al. despite the lack of the *spoVF* locus, which encodes a DPA synthetase [32]. Orsburn et al. has described that the *spoVF* locus is also absent in certain clostridia and that an electron transfer flavoprotein may compensate for DPA generation [33]. Therefore, *spoVF* may not be a strict requirement for DPA production and endosporulation may infrequently encompass genera from *Actionbacteria* and may not be restricted solely to low G+C% bacteria. Future studies should involve mechanistic based experiments to determine if DPA production in *MAP* depends upon an electron transfer flavoprotein. It is possible that novel morphotypes of *MAP* may not be strict endspores and are more reminiscent of *Streptomyces spp.* spore structures.

### Sporulation in MAP is reproducible and consistent in a different culture, medium and multiple strains

**Figure 3:**
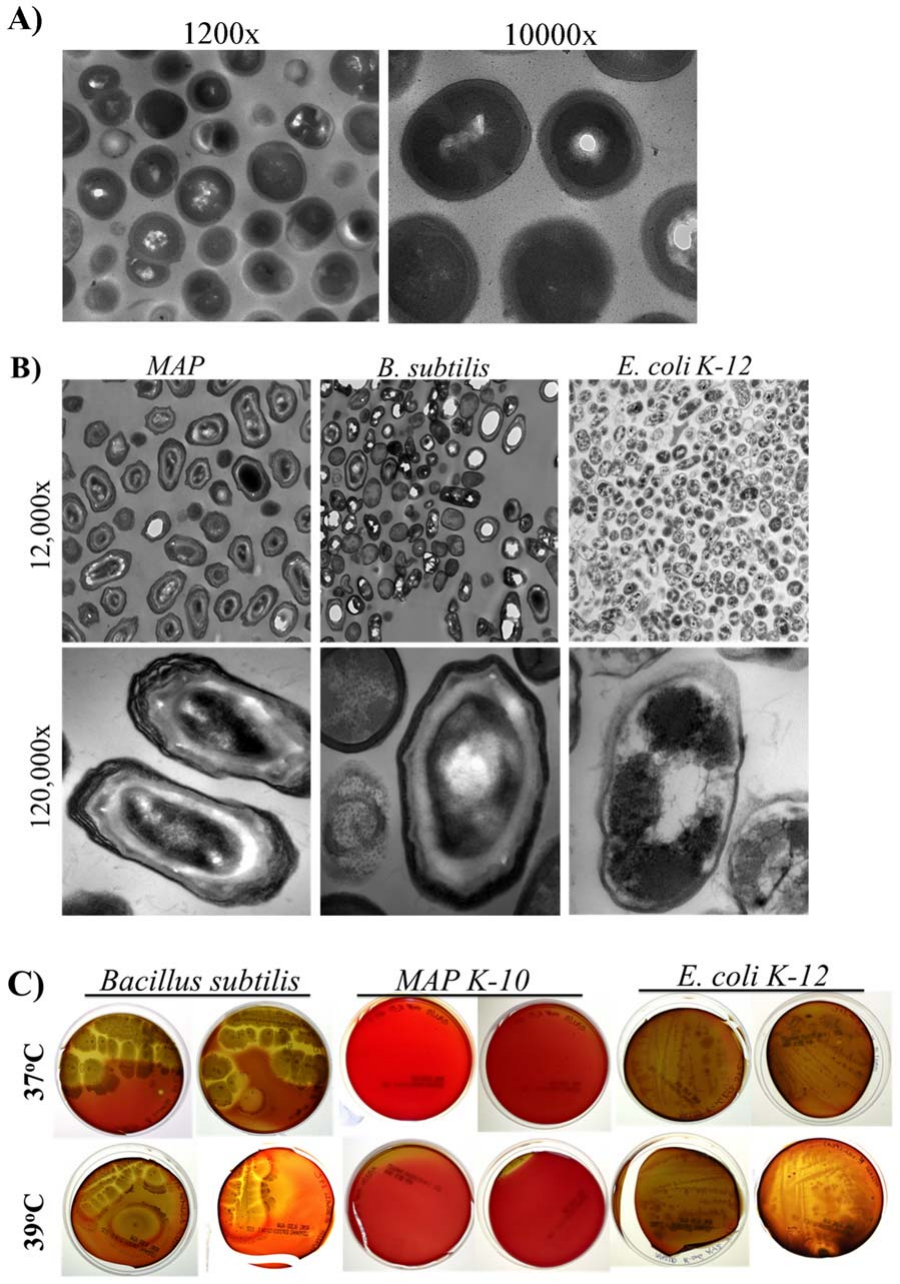
Sporulation is reproducible on traditional sporulation medium. Spore enrichment was independently conducted by the National Animal Disease Center (Ames, IA) using a separate *MAP K-10* culture inoculated on A–K agar. *MAP K-10* year old MB7H9 broth culture, *B. subtilis* and *E. coli K-12* were inoculated on B) Potato extract agar with mycobactin J at 37°C and 39°C. *MAP K-10* growth was observed after two weeks of incubation at 39°C in comparison to overnight growth of *B. subtilis* and *E. coli K-12* controls. Biomasses were collected similarly to A–K cultures and processed for TEM (B). *MAP K-10* TEM images showed similar structures as those observed in **Figure 2**. Furthermore, biomasses were streaked on blood agar and incubated at 37°C and 39°C for 4 weeks to confirm purity (C). *MAP* K-10 failed to grow for the entire duration of incubation in comparison to *B. subitlis* and *E. coli K-12* controls.

**Figure 4:**
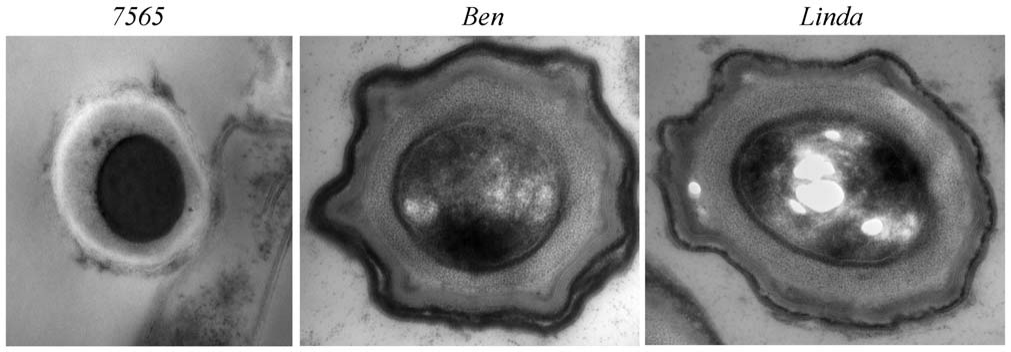
Sporulation occurs in multiple *MAP* strains. *MAP* strains *7565*, *Ben* and *Linda* were inoculated on A–K agar. Biomasses were collected and processed for TEM. All strains show characteristic spore structures.

Our enrichment technique using A–K agar was validated by another laboratory (National Animal Disease Center (NADC), USDA) using a separate MB7H9 liquid culture of *MAP K-10* (Figure 3A). TEM images of *MAP K-10* showed characteristic spore features (e.g. condensed core and coat layers) (Figure 3A). Since A–K agar is not a typical method used to induce sporulation from bacterial cultures, we included spore enrichment using potato extract agar (PEA) using a described method (Figure 3B) [34]. Again, year old liquid culture of *MAP K-10* was capable of producing spore-like morphotypes and displayed identifiable spore structures (Figure 3B). *B. subtilis* and *E. coli K-12* were included as positive and negative controls, respectively (Figure 3B). *MAP K-10* PEA spore-like structures were free of contamination as determined by the absence of growth on blood agar (Figure 3d). Furthermore, we sought to determine if other *MAP* strains were capable of sporulation. A–K enrichment of and TEM visualization showed that *MAP* 7565 (sheep strain), *Ben* (human strain) and *Linda* (human strain) sporulated (Figure 4). Both *Ben* and *Linda* strains were isolated from patients with Crohn's disease [35], [36]. Although controversial, the presence of *MAP* (either in blood or intestinal tissue) has been shown to be associated with Crohn's disease but an etiological link between the two remains to be established [37], [38], [39].

### MAP spore-like forms survive heat treatment and are positive for MAP 16SrRNA and IS900

**Figure 5:**
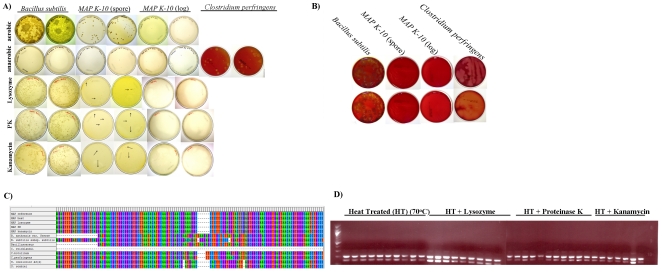
*MAP* morphotypes survive 70°C and are positive for *MAP 16SrRNA* and *IS900*. (A) *MAP K-10* log phase and spores, *B. subtilis* and *C. perfringens* were heat treated at 70°C for 30 min and subsequently treated with 2% lysozyme, PK, or kanamycin. Heat treated samples were plated on MB7H9 or blood agar and incubated at 37°C under aerobic or anaerobic conditions. (B) Heat treated cultures were plated on blood agar to determine growth of any contaminates. (C) *16SrRNA* sequences of germinated heat treated *MAP* spores compared to reference sequences from *MAP*, *Bacillus* spp., *Streptomyces* spp. *and Clostridium* spp.. Ten colonies from each plate were selected for sequences. Sequences shown are a consensus from the ten colonies. (D) *IS900* duplex PCR of germinated heat treated *MAP* spores.

To be considered a true spore former, isolated novel *MAP* morphotypes must survive exposure to heat and be capable to germinate given a nutrient source [26]. We heat treated *MAP K-10* spores at 70°C and 90°C for 30 min in addition to 2% lysozyme, proteinase K (PK), kanamycin and anaerobic exposure. Heat treatment served two purposes to: 1) determine temperature threshold for survival and 2) eliminate any remaining vegetative cells such that the re-grown culture only originated from spores. Both lysozyme and PK are typically used as a standard DNA extraction protocol that functions by damaging the cell wall of vegetative *MAP* cells, which causes bacterial lysis. *MAP K-10* spores survived exposure to 70°C but not at 90°C (Figure 5a). Heat exposed spores treated in combination with either lysozyme, PK or kanamycin were capable of re-growth due to the coat layer resistance to these enzymes (Figure 5a). Incubation under anaerobic conditions was included to rule out *Clostridium spp.* contamination. Exposure to anaerobic conditions post heat treatment failed to produce any visible growth (Figure 5a). Contamination during heat treatment was also eliminated as can be observed by the absence of growth on blood agar plates (Figure 5b). Ten colonies from each heat treated *MAP* plate (70°C alone/and+lysozyme, +PK, +kanamycin) were selected and submitted to *16SrRNA* sequencing. Sequencing results showed that all colonies were positive for *MAP 16SrRNA* (Figure 5c). These colonies were also positive for *MAP-*specific *IS900* (Figure 5d). *MAP* spore survival post exposure to 70°C may in fact not be extremely surprising since many studies have shown the presence of *MAP* as a food contaminant in pasteurized (also treated at 70°C) milk, cheeses and yogurt [40], [41], [42], [43]. It is currently unknown if the *MAP* found in these dairy products may exist in a spore or spore-like state. It is important to note that *MAP* is hypothesized to be one potential trigger or causative agent for Crohn's disease (CD) onset [37], [38], [39], [44]. Several studies indicate that the gross pathology of JD and CD are similar, such as the thickened intestinal mucosa and transmural inflammation [36], [45], [46]. It is proposed that *MAP* survival in pasteurized dairy products may serve as a vehicle for *MAP* infection in a subset of CD patients [41], [47], [48]. If *MAP* does survive pasteurization as a spore, this may result in an important finding and further understanding of *MAP*'s potential role in public health.

### Dormant MAP cultures express elevated transcript levels of sporulation related genes

**Figure 6:**
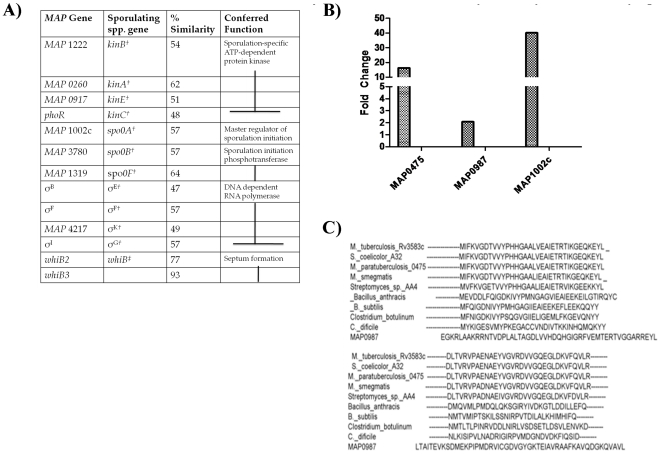
Dormant *MAP* cultures upregulate spore-related transcripts. (A) A BLAST comparison and reciprocal BLAST searches were conducted between known sporulation genes from *Bacillus spp.^†^* and *Streptomyces spp.^‡^* against *MAP*. Percent similarity was determined by protein alignment. (B) Quantitative real-time PCR was performed on dormant *MAP* cultures to determine the presence of *carD (MAP0475* and *MAP0987*) and *spo0A* (*MAP1002c*). All three genes are upregulated in comparison to log-phase *MAP K-10* culture. All samples were conducted in triplicate. C) Multiple sequence alignment of selected CarD proteins. CarD has recently been shown by Stallings et al. to be necessary component of stringency regulation in mycobacteria. Other studies indicate that the stringency response is also necessary for the initiation of sporulation. A multiple sequence alignment of CarD amino acid sequences from mycobacteria and sporulating bacteria was conducted using CLUSTALW.

In addition to spore visualization, we have identified a number of mycobacterial candidate genes corresponding to those in the sporulation pathway of several *Bacillus* and *Streptomyces* species (Figure 6a). We show that *MAP 1002c*, which has a 57% similarity to the sporulation response regulator *spo0A* in *Bacillus spp.*, has a 40-fold increase expression in dormant *MAP K-10* compared to respective log-phase culture (Figure 6b). Other studies have also noted the presence of spore related genes in *M. tuberculosis* and other mycobacterial species [49].

Research investigating *Bacillus spp.* and *S. coelicolor* A3(2) morphology indicate that the stringent response is essential to robust spore production [50], [51]. A recent study by Stallings et al. showed that the mycobacterial gene, *carD*, is an essential regulator of the stringent response that is also found in a number of sporulating bacteria that downregulates rRNA by binding onto the β-subunit of RNA polymerase (RNAP) in response to nutrient starvation and oxidative stress (Figure 6c) [52]. Transcript levels in dormant *MAP* culture show a 15 and 2-fold upregulation of *MAP 0475* and *MAP 0987(carD* orthologues), respectively (Fig. 6b). The presence and absence of orthologous genes alone is unlikely to shed any deeper understanding of the sporulation process in mycobacteria but will require the addition of an evolutionary systems biology approach [53]. In other words, functional assays on identified genes are necessary to assess their impact on the generation of this new *MAP* morphotype. Evolutionary systems biology, which focuses on the changing relationships between genes and gene products, will reveal developmental networks that may include regulatory molecules and feed-forward networks. Ongoing studies in our laboratory are seeking to create knock-out and knock-in mutations of genes identified in Table 1 to determine what role if any they may have in formation of the *MAP* spore-like morphotype.

**Table 1:** Primers used in this study.

Gene and direction	Sequence
*IS900, L1* ±	CCCGTGACAAGGCCGAAGA
*IS900, L9* ±	CGGCCCTGGCGTTCCTATG
*IS900, 900 R* ŧ	ACGCTGTCTACCACCCCGCA
*spoIVA, F*	AAATCGGCACACGAAAAGTC
*spoIVA, R*	TGCCAATACCGGGATATCAT
*Clostridium 16SrDNA, F* Ψ	AAAGATGGCATCATCATTCAAC
*Clostridium 16SrDNA, R* Ψ	TACCGTCATTATCTTCCCCAAA
Universal *16SrRNA, F*	AGAGTTTGATCCTGGCTCAG
Universal *16SrRNA, R*	GGGTGGATCCTCCTT
*MAP1002c, F*	CGGGTGTGGAACTACGACTT
*MAP1002c, R*	TCTTCTTCCTCAGGTACGAGATGT
*MAP0475, F*	GACAAGGTATTCCAGGTGCTG
*MAP0475, R*	CTCGGCGACCTTGTTGAC
*MAP0987, F*	GCACGACGGCATCGTTAT
*MAP0987, R*	GTCAAGTCCGTCCGTCTCGGTGA

± Motiwala, A.S., et al. *Molecular epidemiology of Mycobacterium avium subsp. paratuberculosis: evidence for limited strain diversity, strain sharing, and identification of unique targets for diagnosis.* J Clin Micobiol, 2003. **41**(5):2015–26.

ŧ Bull, T.J., et al. *Characterization of IS900 loci in Mycobacterium avium subsp. paratuberculosis and development of multiplex PCR typing.* Microbiology, 2000. **146**:3285.

Ψ Wang, R.F., et al. *A 16S rDNA-based PCR method for rapid and specific detection of Clostridium perferingens in food.* Mol Cell Probes,m1994. **8**(2): 131–7.

### MAP spores retain infectivity and germinate into acid-fast bacilli during exposure to bovine monocyte derived macrophages (MDMs)

**Figure 7:**
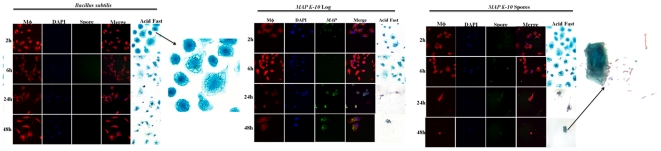
*MAP* spores retain infectivity and germinate into acid-fast bacilli in a bovine MDM model. *MAP* spores, *MAP* log-phase and *Bacillus subtilis* spores were allowed to infect MDMs for 0.5, 2, 6, 24, and 48 h p.i.. *MAP* spores readily infected MDMs and germinated by 24 h p.i.. Upon 48 h p.i., MDMs were lysed and *MAP* spores successfully germinated into acid fast bacilli.

Previous studies have reported that *MAP* can readily be isolated in soil and aquatic environments, which may come into contact with animals and serve as transmission routes [15], [54], [55], [56], [57], [58]. For example, livestock manure stored in liquid lagoons is often applied to agricultural land and *MAP* may persist within this environment upwards of 175 days [59]. Therefore, we asked the question if *MAP* spore-like forms could transmit and maintain infection in a well-developed bovine monocyte derived macrophage (MDM) model. Both *MAP* and *B. subtilis* spores are readily phagocytosed by MDMs; however, *B. subtilis* spores are cleared within 6 h p.i. (post infection) (Figure 7). All vegetative cells were lysed as stipulated in materials and methods. *MAP* spores are maintained within MDMs and germinated by 24 h (Figure 7). Spore germination into developed bacilli show strong acid-fast staining, which is a major diagnostic feature of mycobacteria. Furthermore, the progression of infection with *MAP* spores was enhanced when compared to the *MAP* log-phase control and MDMs lysed at 48 h p.i. (Figure 7). These data combined with heat resistance suggest that sporulation in *MAP* may aid and impact the rate of transmission and consequently establishment of infection in host species.

### Conclusions

This is the first study to identify and describe a new *spore-like* morphotype in *MAP* associated with nutrient starvation. We propose that *MAP* may utilize sporulation as a mechanism to persist in unfavorable conditions such as those encountered in soil and aquatic environments. *MAP* may also commit itself to a spore-like state to survive the pressures applied by pasteurization and thereby provide one explanation for *MAP* detection in commercialized dairy products. Although significant strides have been made, especially within the last ten years, in understanding mycobacterial persistence, it continues to be fraught with ambiguities and dissension. The findings by Ghosh et al., which identified spores in *M. marinum*, and those presented in this paper for *MAP* are difficult to grapple with as they defy key concepts and change our perceptions of persistence, dormancy and transmission for *MAP*. This new *MAP* morphotype or spore readily invaded bovine MDMs, germinated and developed into acid-fast bacilli. More importantly, enrichment and isolation of this new morphotype was independently conducted by a second laboratory (NADC) using a separate *MAP* culture grown at that facility. Concerns are raised due to the similarities of spore formation in *MAP* and *Bacillus* given widely divergent genera. However, 1) certain species of *Streptomyces*, another genus of the *Actinobacteria*, are capable of endospore formation at suitable conditions and 2) DPA is also present in *Streptomyces* despite the lack of *spoVF* operon. Identification of unique *MAP* spore coat proteins as well as the cues leading to sporulation may aid in future diagnostics for food and environmental safety. Further studies are needed to examine the role of this newly described *MAP* morphotype in soil and aquatic environments as well as post pasteurization in dairy products utilizing the above aspects to assess impact in transmission and persistence.

## Materials and Methods

### Bacterial and MDM Cell Culture

Dormant cultures of *MAP* strains *K-10*, *7565*, *Ben* and *Linda* were grown at 37°C in MB7H9 broth supplemented with oleic acid, albumin, dextrose and catalase (OADC) enrichment and mycobactin J (2.0 mg/L) (Allied Monitor, Fayette, MO) for one year without agitation. *MAP* cultures were determined to be free of contamination by absence of growth on BHI agar at 37°C and 39°C. *Bacillus subtilis* and *Escherichia coli K-12* were cultured in Luria-Bertani (LB) broth at 37°C with shaking at 150 rpm. *Clostridium perfringes* was grown on blood agar at 37°C in an anaerobic chamber. Mitsubishi anaeropacks (Fisher Scientific, Pittsburgh, PA) used for the anaerobic chamber were changed every 24 h.

Peripheral blood was collected from the jugular veins of two JD free cattle (542 and 2170) at the University of Minnesota's dairy barn and teaching facility. Monocyte derived macrophages (MDMs) were elutriated using an established protocol [60]. All cattle work performed was in concordance with the institutional guidelines and approved animal care and use protocols at the University of Minnesota.

### Spore-like Morphotype Isolation

Approximately 250.0 µL of dormant cultures of *MAP* (*K-10, 7565, Ben* and *Linda*), log-phase *B. subtilis* (O.D._570_ = 0.5) and log-phase *E. coli* (O.D._600_ = 0.5) were cultured separately on Arret-Kirshbaum (A–K) sporulation agar (BD, Franklin Lakes, NJ) at 37°C and 39°C for either 72 (*MAP*) or 24 h (*B. subtilis* and *E. coli*). A time point of 72 h for *MAP* cultures were selected based on the time required to observe *MAP* growth on A–K plates. *MAP* cultures were also grown on Potato Extract Agar (PEA) at 39°C until growth was achieved (2 weeks) [34]. Upon completion of incubation times, A–K and PEA agar plates were allowed to rest at room temperature (RT) for 48 h. Biomasses were collected in autoclaved distilled water (dH_2_O) and incubated at RT for 72 h.. Spores were washed 3× in 1× phosphate buffer saline (PBS), centrifuged at 13,000 rpm for 10 min to sediment and resuspended in 20.0 mL of autoclaved dH_2_O. *C. perfringes* spores were enriched by incubation with Duncan and Strong broth at 37°C for 24 h under anaerobic conditions. All spore samples were heat treated at 70°C for 30 min to lyse any vegetative cells. All spore samples were differentially stained with malachite green/safranin and visualized on an Olympus IX70 inverted fluorescence microscope (Olympus, Center Valley, PA).

### DNA Extraction and Polymerase Chain Reaction (PCR)

DNA was extracted from 500.0 mg (wet weight) *MAP K-10* log-phase (O.D._600_ = 0.5), dormant cultures and spores by incubating samples for 10 min at 37°C with 10% sodium dodecyl sulfate (SDS) followed by homogenization in a mini-bead beater (Roche) using 0.2 mL of 0.1 mm sterile RNase free zirconium beads (Biospec, Bartlesville, OK) for 4 min. DNA samples were cleaned using PE buffer and spin columns (Qiagen, Valencia, CA). *B. subtilis* DNA was isolated from subcultures grown for 16 h at 37°C using the QIAamp DNA mini kit (Qiagen, Valencia, CA) per manufacturer's instructions. *Clostridium perfringens* DNA was generously provided by Arpita Ghosh (University of Minnesota). All DNA samples were checked for purity and concentration using the 260/280 ratio provided by the NanoDrop sample retention system (Thermoscientific, Wilmington, DE). All primers with the exception of *Clostridium 16SrDNA* [61] were designed using Primer3 software (http://frodo.wi.mit.edu/primer3/) (Table 1). PCR was conducted using Hot-Start Taq (Denville, Metuchen, NJ) per manufacturer's instructions. All *MAP* DNA reactions contained 5% dimethyl sulfoxide (DMSO). The following cycling programs were used for the corresponding genes: ***IS900*** 95°C for 15 min., 94°C for 15 s, 58°C for 20 s, 72°C for 20 s and 72°C for 7 min. for 35 cycles, ***spoIVA*** 94°C 15 min., 94°C 15 s, 51.5°C for 15 s, 72°C for 30 s, and 72°C for 1 min. for 35 cycles, and *Clostridium* ***16SrDNA*** 94°C for 15 min., 94°C for 15 s, 52°C for 15 s, 72°C for 30 s and 72°C for 1 min for 35 cycles.

### Transmission Electron Microscopy (TEM)

TEM ultrastructural imaging was conducted on MAP log-phase, dormant and isolated spore cultures using the methods described by Ghosh et al. [10]. Dormant cultures and A–K and PEA biomasses of *MAP* strains *K-10, 7565*, *Linda* and *Ben* were centrifuged at 3,000 rpm and supernatants were discarded. Pellets were fixed with 2.5% glutaraldhyde in 0.1 M sodium cacodylate buffer overnight at 4°C. Samples were washed 3× using 0.1 M sodium cacodylate buffer and post fixed in 1% osmium tetroxide in 0.1 M sodium cacodylate buffer reduced with ferroyanide and washed 3× in autoclaved water. After a series of acetone dehydration, all samples were infiltrated with 1∶2, 2∶1 resin∶acetone mixtures and 3 100% resin. Samples were embedded and cured at 60°C for 48 h and later visualized using TEM.

### DPA Assay

DPA was detected using a previously reported colorimetric assay [29]. Wet pellet weight of 250.0 mg was used for each bacterial sample. Autoclaved and “intact” mycobactin J (250 µg/mL) were used as controls. DPA concentrations were calculated based on a DPA (MP Biomedicals, Riverside CA) protein concentration curve. DPA assay was performed six times and all samples were conducted in triplicate with duplicate technical replicates. Figure 2C shows a representative experiment.

### Heat Treatment

*MAP K-10* spores, *B. subtilis* spores and *C. perfringes* spores were heat treated at 70°C and 90°C for 30 min. Additional treatments post heating included exposure to 2% lysozyme at 37°C for 10 min, PK (100 µL of 2 mg/mL) digestion at 37°C for 10 min, kanamycin (50 µg/mL) at 37°C for 2 h and anaerobic exposure at 37°C for 3 weeks. To ensure that *MAP* cultures were devoid of contaminants post heat treatment, all samples were grown on blood agar at 37°C for 4 weeks. Ten colonies were selected from each *MAP* heat treatment plate and re-grown in MB7H9 broth at 37°C for 10 weeks. DNA was extracted from re-grown cultures and sequenced for *16SrRNA* (Table 1). The universal *16SrRNA* cycling program is as follows: 94°C for 15 min., 94°C for 15 s, 54°C for 15 s, 72°C for 30 s and 72°C for 1 min for 30 cycles. *16SrRNA* sequences were analyzed using Sequencher (Gene Codes Corporation, Ann Arbor, MI) and aligned using MEGA software (http://www.megasoftware.net/) [62]. Colonies were further submitted to *IS900* PCR. All samples were plated twice and heat treatment experiments were conducted three separate times.

### RNA Extraction and Qt-RT-PCR

1.0 mL of dormant and log-phase *MAP K-10* cultures were centrifuged separately in 1.5 mL eppendorf tubes for 10 min. at 13,000 rpm. Supernatants were decanted and pellets were washed 3× in 1× PBS. 1.0 mL of TRIzol reagent (Invitrogen, Carlsbad, CA) was added to each sample and allowed to incubate at room temperature for 5 min. *MAP K-10* samples mixed with TRIZol were homogenized using 0.3 ml of 0.1 mm sterile RNase-free zirconium beads for 4 min. in the MagNa Lyser system (Roche, Basel, Switzerland). RNA was extracted following TRIzol protocol. RNA was subsequently treated with TurboDNase (Ambion, Austin, TX) for 30 min at 37°C. All samples used had a 260/280 ratio of at least 1.9 as measured by NanoDrop sample retention system (Thermoscientific, Wilmington, DE). Primers used for Qt-RT-PCR were designed using Primer3 software (http://frodo.wi.mit.edu/primer3/) (Table 1). Qt-RT-PCR analysis was conducted on 50.0 µg of purified dormant or log-phase *MAP K-10* culture combined with the Quantifast one-step RT-PCR reagents (Qiagen, Valencia, CA) using the Lightcycler 480 II (Roche, Basel, Switzerland) programmed for the following: 50°C for 10 min, 95°C for 5 min, 95°C for 10 s, 60°C for 30 s for 40 cycles. Fold change was calculated using the 2^−ΔΔCt^ method. All samples were conducted in triplicate.

### Spore Invasion Assay

Bovine MDMs in RPMI containing 2% autologous serum were seeded at 2.0×10^4^ cells/mL in a 24 well plate containing 1.0 No. 1.5 thickness glass coverslips and allowed to adhere for 2 h at 37°C in humidified incubator containing 5% CO_2_. Following incubation, MDMs were washed 3× in 1× Dulbecco's phosphate buffer saline (D-PBS) to remove non-adherent cells and medium was replaced with fresh serum-free RPMI prior to infection. *MAP* K-10 subculture and spores and *B. subtilis* spores were pelleted at 13,000 rpm for 5 min. or 10 min., respectively, washed 2× with warm 1× D-PBS and resuspended in 37°C warmed serum-free RPMI such that a MOI (multiplicity of infection) ratio of 10∶1 was achieved. Serum free RPMI was used to prevent spore germination outside of MDMs. Spores were heat treated at 70°C for 30 min. Subsequently, cultures were vigorously vortexed and allowed to rest for 5 min. at 37°C so that potential clumps would settle to the bottom of the tube. An 18.5 gauge syringe needle was used to repeatedly draw the upper three-fourths of the newly suspended *MAP* cultures to disperse any remaining clumps. MDMs were separately infected with upper three-fourths of RPMI-*MAP*/spore cultures for 2 h at 37°C in humidified incubator containing 5% CO_2_, rinsed 3× with 1× D-PBS and resuspended in RPMI containing 2% autologous serum for the following post infection (p.i.) time points: 0, 0.5, 6, 2, 24, and 48 h. Upon completion of post infection time points, MDMs were rinsed 3× in D-PBS and fluorescently or acid-fast stained for visualization. All time points were conducted in triplicate.

### Cell Staining

Fluorescent staining was conducted as stipulated by Lamont et al. [16] with the exception that log-phase and spore cultures were pre-stained for 30 min in 2.0 mg/mL of 5-carboxyfluorescein diacetate (CFDA) (Sigma-Aldrich, St. Louis, MO) at 37°C. Fluorescent images were visualized and collected as a Z-series (step size: 1.0 µm) using DAPI, FITC and Cy5 lasers on an Olympus Fluoview 1000 upright confocal microscope (Olympus, Center Valley, PA). Joint acid-fast images were stained using a modified Zeil-Neelsen protocol (Trend Laboratories Inc., Atlanta, GA) and imaged on an Olympus IX70 inverted fluorescence microscope.
